# In silico identification of novel SARS-COV-2 2′-O-methyltransferase (nsp16) inhibitors: structure-based virtual screening, molecular dynamics simulation and MM-PBSA approaches

**DOI:** 10.1080/14756366.2021.1885396

**Published:** 2021-03-09

**Authors:** Mahmoud A. El Hassab, Tamer M. Ibrahim, Sara T. Al-Rashood, Amal Alharbi, Razan O. Eskandrani, Wagdy M. Eldehna

**Affiliations:** a Department of Pharmaceutical Chemistry, Faculty of Pharmacy, Badr University in Cairo (BUC), Badr City, Egypt; b Department of Pharmaceutical Chemistry, Faculty of Pharmacy, Kafrelsheikh University, Kafrelsheikh, Egypt; c Department of Pharmaceutical Chemistry, College of Pharmacy, King Saud University, Riyadh, Saudi Arabia

**Keywords:** SARS COV-2 2′-O-methyltransferase (nsp16) Inhibitor, 3D pharmacophore, molecular dynamics, MM-PBSA calculations, COVID-19 therapies

## Abstract

The novel coronavirus disease COVID-19, caused by the virus SARS CoV-2, has exerted a significant unprecedented economic and medical crisis, in addition to its impact on the daily life and health care systems all over the world. Regrettably, no vaccines or drugs are currently available for this new critical emerging human disease. Joining the global fight against COVID-19, in this study we aim at identifying a potential novel inhibitor for SARS COV-2 2′-O-methyltransferase (nsp16) which is one of the most attractive targets in the virus life cycle, responsible for the viral RNA protection *via* a cap formation process. Firstly, nsp16 enzyme bound to Sinefungin was retrieved from the protein data bank (PDB ID: 6WKQ), then, a 3D pharmacophore model was constructed to be applied to screen 48 Million drug-like compounds of the Zinc database. This resulted in only 24 compounds which were subsequently docked into the enzyme. The best four score-ordered hits from the docking outcome exhibited better scores compared to Sinefungin. Finally, three molecular dynamics (MD) simulation experiments for 150 ns were carried out as a refinement step for our proposed approach. The MD and MM-PBSA outputs revealed compound **11** as the best potential nsp16 inhibitor herein identified, as it displayed a better stability and average binding free energy for the ligand-enzyme complex compared to Sinefungin.

## Introduction

Since 11th March 2020, COVID-19 caused by sever acute respiratory syndrome (SARS-COV-2) virus was declared by the WHO as a pandemic disease. The virus is extremely dangerous affecting more than 200 countries with 46 202 728 confirmed cases and 1,197,739 deaths by the 31th of October 2020. The available treatment protocol for COVID-19 infection is limited to a few drugs that at their maximum efficacy may only reduce the symptoms of the infection^1^. Thus, it is an urgent need to develop a specific treatment for the COVID-19 infection using various drug discovery means. There are many approved direct acting antiviral agents (DAA), which have significantly reduced or diminished the viral load and have achieved great therapeutic outcomes in the treatment of viruses such as Hepatitis Virus B, C, or AIDS. Each of these DAAs has a specific target in the virus life cycle for instance, Simeprevir acts on HCV NS3 protease while Elvitegravir acts on HIV integrase^2^^,^^3^. So, developing a specific potent DAA for COVID-19 infection could be achieved only if full understanding of the function and structural features of essential targets is provided.

SARS-COV2 transmission could be conveyed through different modes, e.g. mainly through respiratory droplets, and like other respiratory viruses when exposing to sneezing or coughing from COVID-19 patients, through faces, and close contact less than 2 metres^4^. The crown-like virus has a genome of ∼30000 nucleotides in length which encodes several proteins. Four of them are structural proteins namely, Nucleocapsid (N) protein, Membrane (M) protein, Spike (S) protein and Envelop (E) protein and the rest of the encoded polyprotein is non-structural proteins (nsp) that are essentials in the virus replication cycle^5^^,^^6^.

The life cycle of the virus starts after attaching its spike protein to the human angiotensin converting enzyme 2 (ACE2) receptors present at the surface of numerous cells like those of lungs and GIT. Then, the fusion peptide is released after proteolytic cleavages of the Spike protein by host proteases. This is followed by a cascade of cellular processes that end by virus entry into the cytoplasm. After that, the virus is uncoated releasing its single-stranded RNA genome into cytoplasm where the replication and transcription take place by the aid of the virus several non-structural proteins. Finally, the resulting proteins from the replication and transcription processes are assembled into new virions ready to infect new cells^5–7^. Many essential targets in the described life cycle had been proposed as potential for developing specific DAAs for COVID-19 infection such as the non-structural protein 12 (nsp12); RNA Dependent RNA Polymerase (RdRp) which is essential in replicating the virus genome. Also, the 3C-like protease (3CL^pro^) and Papin like protease (PL^Pro^) are two important targets that play a crucial role in the SARS-COV-2 replication cycle by processing the resulting polyprotein from the transcription stage into functioning subunits. Moreover, many studies had been reported aiming to prevent the virus entry by blocking the attachment between the Spike protein and the human angiotensin converting enzyme 2 receptor (ACE2)^8–10^.

Another promising target that worth detailed mentioning is the SARS COV-2 2′-o-methyltransferase (nsp16), which is an important enzyme for the virus survival. The role of the SARS COV-2 2′-o-methyltransferase (nsp16) is to protect the viral RNA from the cellular innate immunity through participation in the formation of a specific arrangement at the 5′ end of the RNA molecule that consists of *N*-methylated guanosine triphosphate and *C*2′-O-methyl-ribosyladenine. This arrangement is called RNA cap that resembles the native mRNA of the host cells, stabilises the RNA, and ensures effective process of its translation. The cap formation starts when 5′-RNA triphosphatase removes a γ-phosphate from a 5′-triphosphate end of the nascent RNA. Then, to the formed 5′-diphosphate end of RNA, guanylyltransferase attaches a guanosine monophosphate (GMP). At last, two steps of methylation are executed by two distinct enzymes, nsp14 that adds a methyl group at *N*-7 of the GTP nucleobase (*N*-7 methyltransferase) and nsp16 that adds a methyl group at *C*2′-O of the next nucleotide^11^^,^^12^. This process is crucial for RNA stability, preventing its degradation by the host^13^. The process and importance of cap formation revels that SARS COV-2 2′-O-methyltransferase (nsp16) is a very promising target and its targeting could result in effective inhibition of COVID-19 infection^13^^,^^14^. We had successfully reported the ability of Structure-Based Drug Discovery (SBDD) in the identification of novel potential inhibitor for SARS-COV-2 Polymerase enzyme^15^.

**Figure 1.**
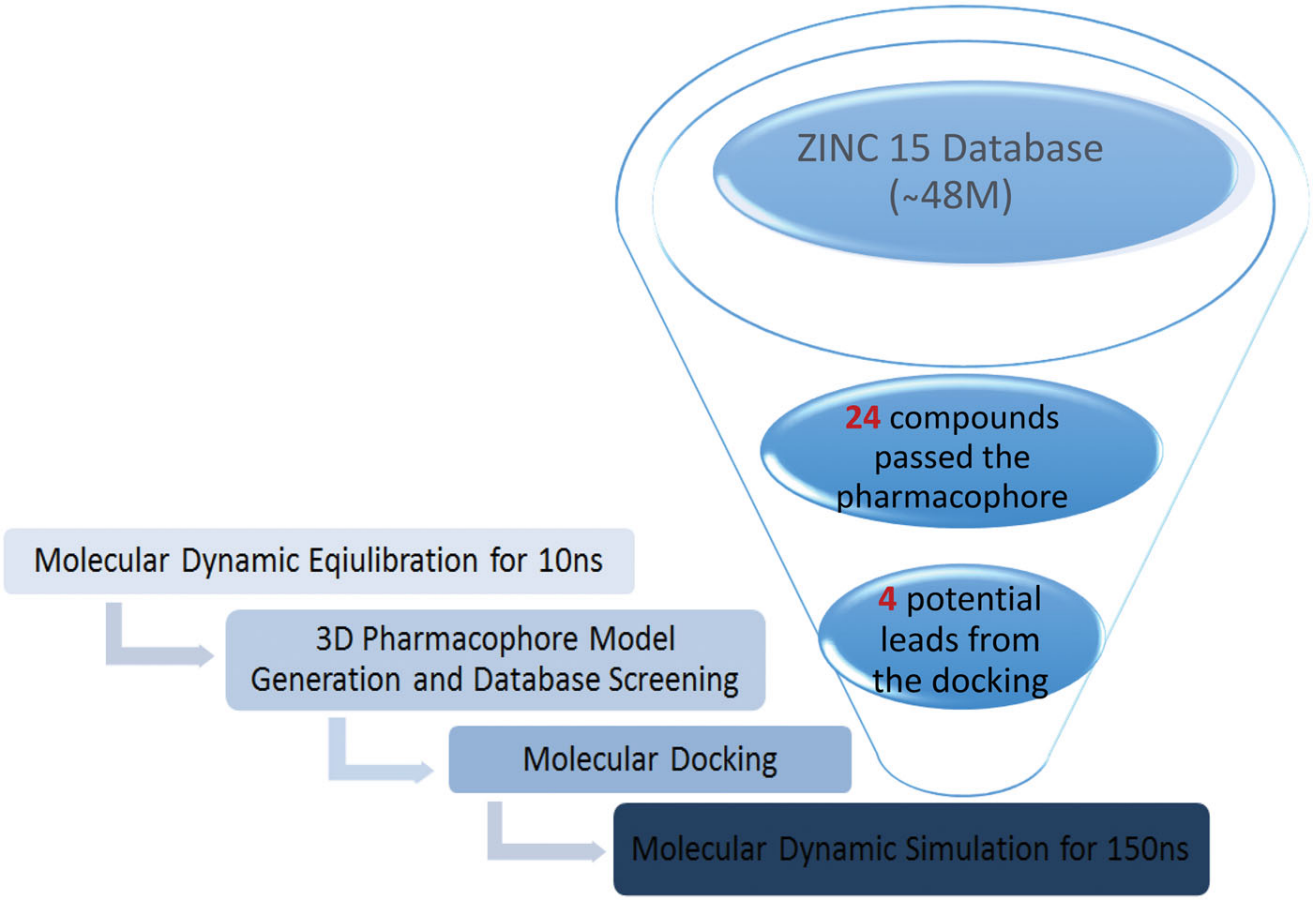
The virtual screening steps and the implemented protocol for the identification of potential inhibitors for SARS COV-2 2′-O-methyltransferase (nsp16).

Herein in this study, we report the application of Structure-Based Virtual Screening (SBVS) strategies with the prime goal of identification of novel potential inhibitors for SARS COV-2 2′-O-methyltransferase (nsp16) utilising integrated Structure-based virtual screening, molecular docking, molecular dynamics simulation and MM-PBSA approaches, as illustrated in (Figure 1).

## Materials and methods

### 3D Pharmacophore generation

**Figure 2.**
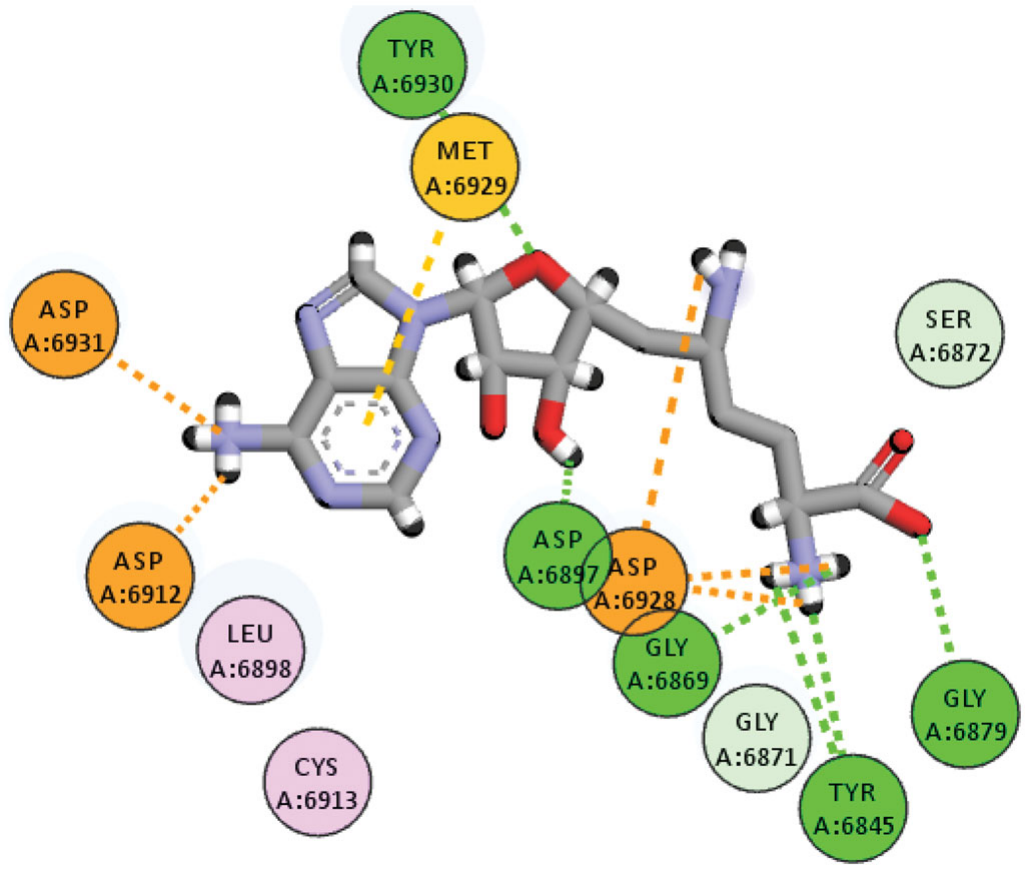
2D Binding of sinefungine within SARS-COV-2 2′-O-methyltransferase binding site after 10 ns of equilibration.

The crystal structure of the SARS COV-2 2′-O-methyltransferase (nsp16) in complex with the pan-methyl-transferase inhibitor Sinefungin was retrieved from the protein data bank; PDB ID: 6WKQ. The retrieved complex was energy minimised by employing the steepest descent minimisation algorithm with a maximum of 50,000 steps and <10.0 kJ/mol force, then the energy minimised complex was equilibrated for 10 ns to elucidate the most stable conformation and to prepare the complex for further *in silico* experiments *see Molecular dynamic section*. The interaction diagram between Sinefungin and the minimised and equilibrated SARS COV-2 2′-O-methyltransferase (nsp16) was generated by Discovery Studio Visualiser 2020 to determine the types and number of the formed interactions, as well as the functional groups in Sinefungin responsible for those interactions (Figure 2). MOE 2019 was implemented to generate 3D pharmacophore features according to the interacting function groups of Sinefungin with its target^16^. Thereafter, the generated pharmacophore was used to screen the ZINC15 database (−48 M compounds) aiming to identify potential potent inhibitors for SARS COV-2 2′-O-methyltransferase (nsp16).

### Database generation and optimisation for pharmacophore screening

The Zinc Drug-like library (nearly 48 million compounds, available at http://zinc.docking.org) was downloaded as smiles files and then converted into single database file with extension mdb by MOE suite (MOE, 2019 (https://www.chemcomp.com)). AMBER14: EHT force field was used for energy minimisation of the generated compound library using steepest descent algorithm until RMS gradient of 0.1 Kcal/Mol/A is reached^16^.

### Virtual screening

A two-stage virtual screening approach, combining pharmacophore-based virtual screening and docking-based virtual screening was employed to identify potential active compounds against nsp16.

### Pharmacophore-based virtual screening

The prepared compounds library of the Zinc database was screened using the constructed 3D pharmacophore model using MOE 2015 pharmacophore wizard^17^^,^^18^. After a series of trials; all the screened compounds were required to match five of the features in the pharmacophore hypothesis. The distance matching tolerance was designated to 2.0 Å. A fitness score was used to rank the database hits based on their RMSD with the hypothesis involving site matching, vector alignments and volume terms. Compounds that passed the pharmacophore filter were further screened through docking-based virtual screening.

### Docking-based virtual screening

This section aims to identify the most potential active compounds resulting from the pharmacophore search as well as predicting their possible mode of binding to the SARS COV-2 2′-o-methyltransferase (nsp16). To provide a rough validation of the docking protocol used, we performed a pose-retrieval docking experiments for the X-ray coordinates of Sinefungin in the binding site of nsp16, for two scenarios, in presence and in absence of water molecules of the binding site. The calculated RMSD values were 0.53 and 0.25 Å in the NW-docking (docking without water) and the W-docking (docking with water) respectively, therefore the water molecules were removed in docking procedure to increase the diversity of selected compounds ^19^ (Figure S1, Supplementary information). AutoDock Vina software was used to conduct the previous step in addition to the successful candidates from the pharmacophore search. MGL tools 1.5.7 was implemented to prepare the equilibrated nsp16, Sinefungin and the successful candidates from the pharmacophore search were converted into pdbqt format; a prerequisite for Vina Autodock software^20^^,^^21^. The active site was determined by generating a grid box sized 24 × 24 × 24 A^0^ surrounding the binding site of Sinefungin. The best candidates were selected for further analysis based on the docking and the interaction diagram generated by Discovery Studio Visualiser^22^.

### Molecular dynamics

We conducted three molecular dynamic simulations experiments to support our concept of design and to validate the predicting binding mode of compound **11**. Two experiments were conducted for SARS COV-2 2′-o-methyltransferase (nsp16) complex with Sinefungin and compound **11**, respectively, while another one was for the free unbound SARS COV-2 nps16. The latest version of GROningen MAchine for Chemical Simulations (GROMACS 2020.3) was employed to conduct the entire MD simulation experiments^23^. The ligand topologies were obtained by the CHARMM General Force Field CGENFF server and converted into the desired gromacs format using the “cgenff_charmm2gmx_py3_nx2.py” script, while the receptor topology was obtained by the “pdb2gmx” script^24^. The generated ligand topologies were rejoined to the processed receptor structure to construct the ligand-protein complex. All the processed complexes were energy minimised under GROMOS96 43a1 force field^25^. After that, those complexes were solvated with a single point charge (SPC) water model to add water molecules to the cubic simulation boxes.

System neutralisation of the net charges was done by adding counter-ions using the “gmx genion” script. Energy minimisation of the unbound enzyme and the two complexes was achieved by employing the steepest descent minimisation algorithm with a maximum of 50,000 steps and <10.0 kJ/mol force. Then, the solvated energy minimised structures were equilibrated with two consecutive steps. Firstly, NVT ensemble with constant number of particles, volume and temperature (310 K) was done for 2 ns followed by NPT ensemble with constant number of particles, pressure and temperature for 8 ns. In the two systems, only the solvent molecules were allowed for free movement to ensure its equilibration in the system while other atoms were restrained. The long range electrostatic interactions were obtained by the particle mesh Eshwald method with a 12 Å cut-off and 12 Å Fourier spacing^26^. Finally, the three well-equilibrated systems (one empty protein and two protein-ligand complexes) were then entered the production stage without any restrains for 150 ns with a time step of 2 fs, and after every 10 ps the structural coordinates were saved to retrieve 15000 frames for each processed complex. The root mean square deviation (RMSD) was calculated from the generated trajectories of the MD simulations as well as the distances of the formed hydrogen bonds between the receptor and the ligands by various scripts of GROMACS.

### MM-PBSA calculation

An important feature of MD simulations and thermodynamic calculations coupling is the ability to measure the binding free energy of a protein-ligand complex. Combining molecular Mechanic/Poisson-Boltzmann Surface Area (MM-PBSA) alongside MD simulations has been reported in successful calculation of the binding free energy of protein and ligand complexes via the application of the following equation:

$${\text{ΔG}}_{\left(\mathrm{Binding}\right)}={\text{G}}_{\left(\mathrm{Complex}\right)}-{\text{G}}_{\left(\mathrm{Receptor}\right)}-{\text{G}}_{\left(\mathrm{Ligand}\right)}$$

Where, G (complex) is the total free energy of the protein − ligand complex and G (receptor) and G (ligand) are total free energies of the isolated protein and ligand in solvent, respectively. The total free energy of any of the three mentioned entities (complex or receptor or ligand) could be calculated from its molecular mechanics potential energy plus the energy of solvation. Thus, the “g_mmpbsa”^27^ package of GROMACS was used perform MM-PBSA calculations through all the MD trajectories.

## Results and discussion

### 3D Pharmacophore

Analysis of SARS-COV-2 2′-O-methyltransferase (nsp16) crystal structure that contains Sinefungine as a co-crystalized ligand revealed numerous numbers and types of interactions between Sinefungine and its corresponding binding domain. Converting all those interactions to 3D pharmacophore features is practically inapplicable and the virtual screening will fail to find any ligand (Figure S2, Supplementary Information). Indeed, not all the formed interactions between a ligand and its target are responsible directly for the ligand activity, otherwise only stable interactions may have the most significant contribution to the activity. Accordingly, the crystal structure was energy minimised and equilibrated for 10 ns to elucidate such stable interactions and then convert them to 3D pharmacophore features. In the equilibrated structure, Sinefungine was able to maintain strong pattern of interaction through seven function groups, (Figure 2).

**Figure 3.**
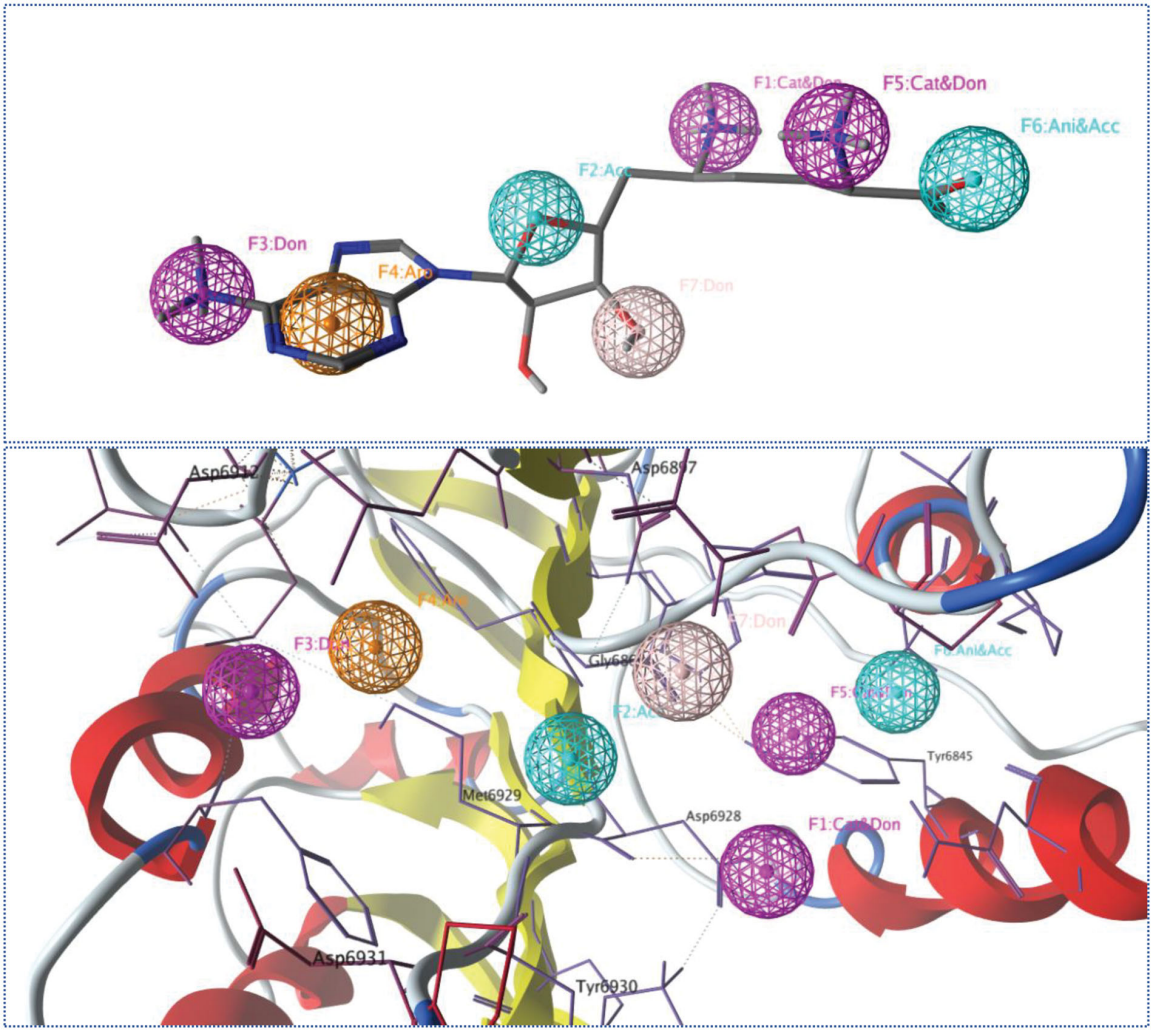
The generated 3D pharmacophore; Blue spheres represent acceptor features, pale pink sphere represents donor features, dark pink spheres represent cationic donor features and orange sphere represents aromatic feature.

The interacting atoms within each functional group in Sinefungine, as well as, the interaction types enabled us to generate a 3D pharmacophore of four features and seven components (Figure 3). Namely, the pharmacophore model consisted of three cationic donor features to attract the negative charge on (Asp6912, Asp6928 and Asp6931), one donor feature to interact with (Asp6897), two acceptor features to interact with (Gly6879 and Tyr6930), and an aromatic feature to interact with (Met6929). The generated pharmacophore was used to screen the ZINC15 drug-like database and the successful compounds were required to match all the features. Unfortunately, no compound was able to match all the features and the same happened when compounds were required to match six of the seven features. Thus, compounds were asked to match at least five of the seven features and this resulted in 24 potential lead compounds (Supplementary Information) that may act as SARS COV-2 2′-O-methyltransferase (nsp16) inhibitors. Thereafter, these potential lead compounds were further evaluated against SARS COV-2 2′-O-methyltransferase (nsp16) *via* molecular docking studies.

### Docking

In Computer-aided drug design studies, docking strategy stands out as one of the most important techniques providing many useful applications, including the prediction of the binding mode between a ligand and its target, ranking a library of compounds based on their docking scores and correlating those scores with potential activity. Also, docking has a valuable role in characterising the effect of certain amino acids mutations on the activity profile of the ligands. Moreover, visualising the interaction images resulting from docking software gives insights and guides for the optimisation of the existing ligands to yield compounds with better affinity. In the current study, docking was implemented to rank the successful 24 candidates from the pharmacophore search, besides predicting the plausible binding mode with their target.

**Figure 4.**
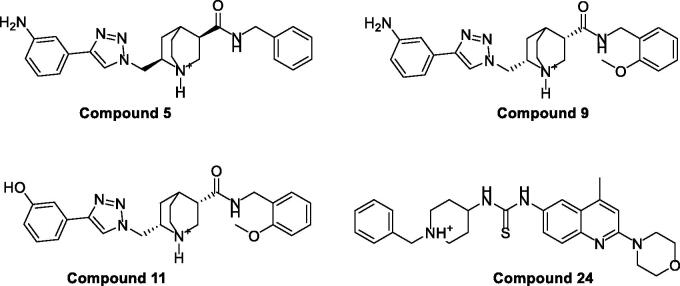
Chemical structures for compounds **5**, **9**, **11** and **24**.

Based on the results obtained from AutoDock Vina, only four compounds (**5**, **9**, **11** and **24**, Figure 4), among the 24 potential leads, were able to achieve better docking score (*S* = −10.1, −10.3, −10.6 and −9.9 Kcal/mol, respectively) than Sinefungine (*S* = −8.5 Kcal/mol), and thus were selected for further analysis.

**Figure 5.**
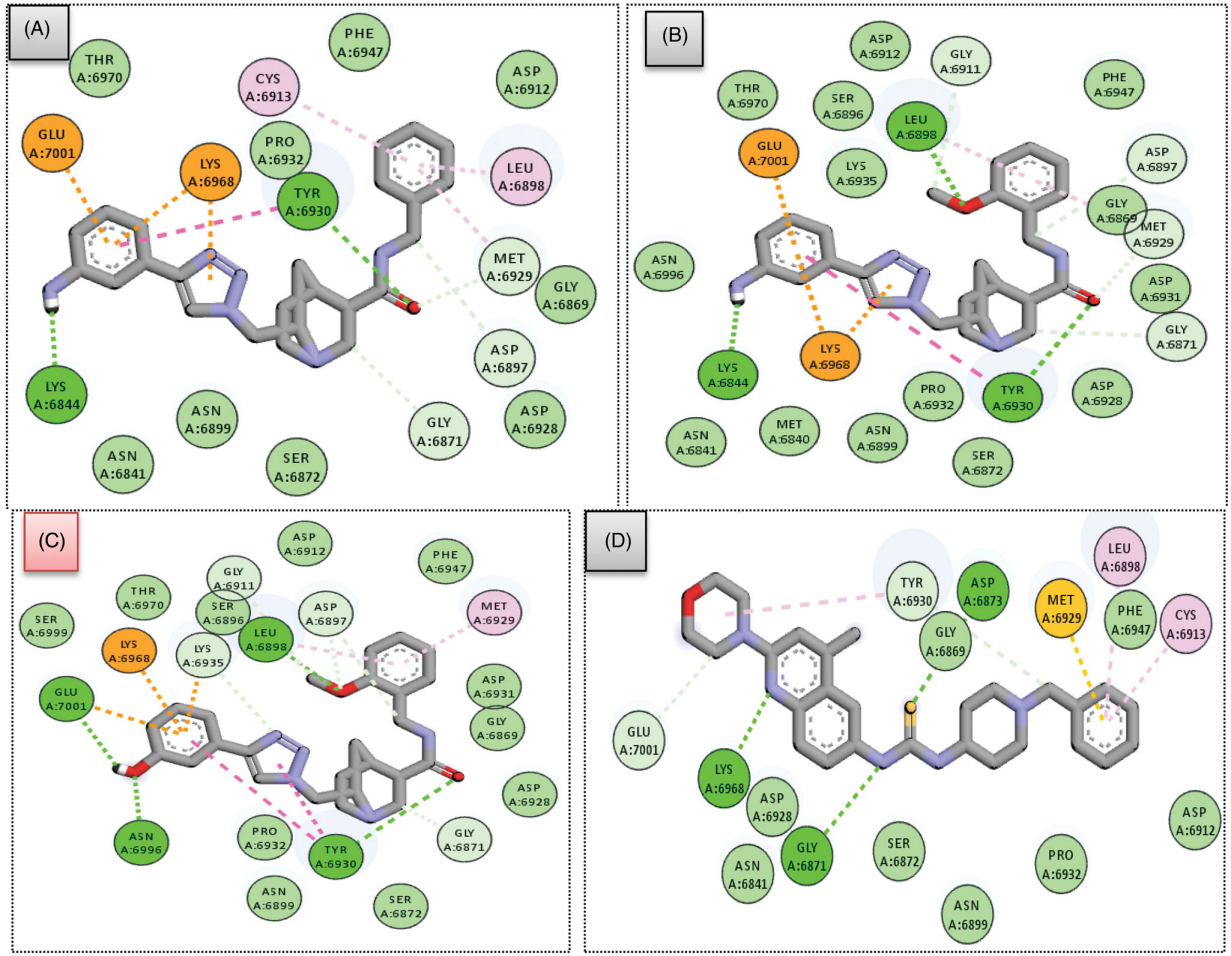
2D interactions of compounds **5** (A), **9** (B), **11** (C) and **24** (D) within SARS-COV-2 2′-O-methyltransferase binding site.

**Figure 6.**
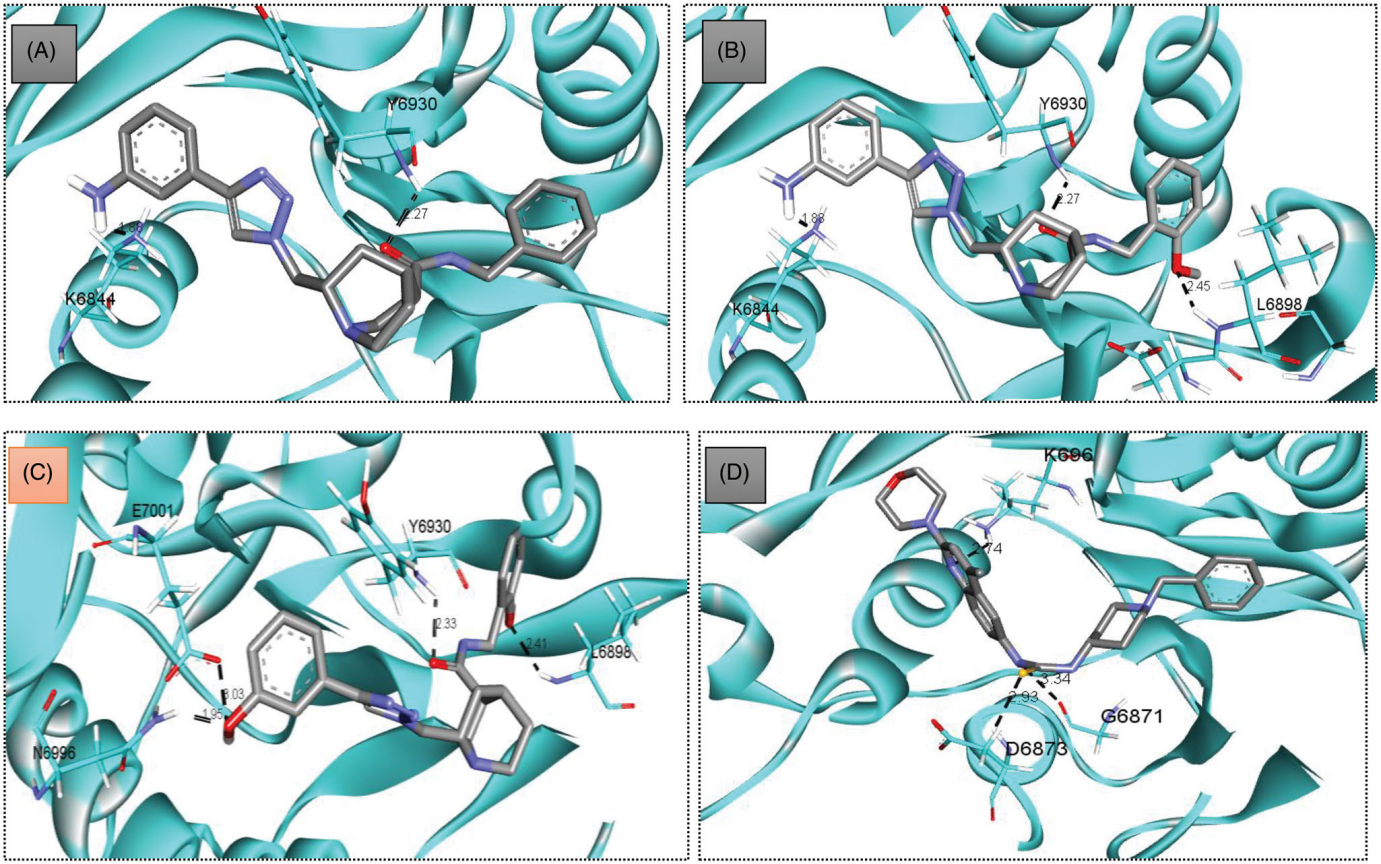
3D representation for inhibitors **5** (A), **9** (B), **11** (C) and **24** (D) displaying their interactions within SARS-COV-2 2′-O-methyltransferase binding site.

Compounds **5**, **9** and **11**, with the same scaffold and similar structures, displayed a similar strong pattern of interaction with their target, which indicates a valid docking approach. On the other hand, compound **24** is based on a different scaffold and thus it was oriented into the receptor in a different manner. In general, the four compounds (**5**, **9**, **11** and **24**) demonstrated a strong binding affinity with many types of interaction with the SARS-COV-2 2′-O-methyltransferase binding site (Figures 5 and 6).

In particular, methoxy-bearing compounds **9** and **11** achieved the best docking score (*S* = −10.3 and −10.6 Kcal/mol, respectively) among the four compounds. They were engaged in two hydrogen bond interactions *via* both methoxy (OCH_3_) and amidic carbonyl (C = O) functional groups with Leu6898 and Tyr6930 amino acids, respectively (Figure 5, 6(B–C)). In addition, the primary amino group (NH_2_) in compound **9** was involved in a hydrogen bond interaction with Lys6844 amino acid, whereas, the hydroxyl group (OH) in compound **11** was able to establish two hydrogen bond interactions with Asn6996 and Glu7001 amino acids.

On the other hand, compound **5** lacks the methoxy group found in compounds **11** and **9**, thus, it was able to form only two hydrogen bonds with Tyr6930 and Lys6844 via its amidic carbonyl (C = O) and primary amino group (NH_2_) functional groups (Figure 5, 6(A)). Furthermore, compound **24** was involved in three hydrogen bond interactions with Gly6871, Asp6873 and Lys6968, yet it came in the last rank (*S* = −9.9 Kcal/mol) as it lacks various hydrophobic interactions achieved by the other three compounds (Figure 5, 6(D)). Table 1 summarises all the bonding interactions and distances for compounds (**5**, **9**, **11** and **24**) within SARS-COV-2 2′-O-methyltransferase binding site.

**Table 1.** Different bonding types and their distances (in Å) for compounds (**5**, **9**, **11** and **24**) within SARS-COV-2 2′-O-methyltransferase binding site

Compound	Energy Score (kcal/mol)	Interactions	Distance
**11**	–10.6	Hydrogen bond with Leu6898	2.41
		Hydrogen bond with Tyr6930	2.33
		Hydrogen bond with Asn6996	1.95
		Hydrogen bond with Glu7001	3.03
		Non-classical carbon Hydrogen bond with Gly6871	3.50
		Non-classical carbon Hydrogen bond with Asp6897	3.39
		Non-classical carbon Hydrogen bond with Asp6897	2.54
		Non-classical carbon Hydrogen bond with Gly6911	3.63
		Non-classical carbon Hydrogen bond with Lys6935	2.98
		Pi-Alkyl interaction with Leu6898	5.05
		Pi-Alkyl interaction with Met6929	4.14
		Pi-Pi interaction with Tyr6930	4.82
		Pi-Pi interaction with Tyr6930	4.87
		Pi-Anion interaction with Glu7001	3.99
		Pi-Cation interaction with Lys6935	3.14
		Pi-Cation interaction with Lys6996	3.84
**9**	–10.3	Hydrogen bond with Leu6898	2.45
		Hydrogen bond with Tyr6930	2.27
		Hydrogen bond with Lys6844	1.88
		Non-classical carbon Hydrogen bond with Gly6871	3.56
		Non-classical carbon Hydrogen bond with Asp6897	3.39
		Non-classical carbon Hydrogen bond with Gly6911	3.68
		Non-classical carbon Hydrogen bond with Met6929	2.70
		Pi-Alkyl interaction with Leu6898	5.07
		Pi-Alkyl interaction with Met6929	4.09
		Pi-Pi interaction with Tyr6930	5.50
		Pi-Anion interaction with Glu7001	3.44
		Pi-Cation interaction with Lys6968	3.17
		Pi-Cation interaction with Lys6968	3.79
**5**	–10.1	Hydrogen bond with Tyr6930	2.27
		Hydrogen bond with Lys6844	1.88
		Non-classical carbon Hydrogen bond with Gly6871	3.52
		Non-classical carbon Hydrogen bond with Asp6897	3.55
		Non-classical carbon Hydrogen bond with Met6929	2.70
		Pi-Alkyl interaction with Leu6898	4.83
		Pi-Alkyl interaction with Met6929	4.21
		Pi-Alkyl interaction with Cys6913	5.49
		Pi-Pi interaction with Tyr6930	5.5
		Pi-Anion interaction with Glu7001	3.44
		Pi-Cation interaction with Lys6968	3.79
		Pi-Cation interaction with Lys6968	3.12
**24**	–9.9	zHydrogen bond with Lys6968	2.74
		Hydrogen bond with Gly6871	3.34
		Hydrogen bond with Asp6873	2.93
		Non-classical carbon Hydrogen bond with Glu7001	3.72
		Non-classical carbon Hydrogen bond with Tyr6930	3.48
		Pi-Alkyl interaction with Leu6898	4.84
		Pi-Alkyl interaction with Tyr6930	5.18
		Pi-Alkyl interaction with Cys6913	4.84
		Pi-Sulfur interaction with Met6929	3.53

### Molecular dynamics

In many computational studies of drug discovery, Molecular dynamic simulations have provided valuable assessment in identification of potential inhibitors for promising targets, studying the nature of macromolecules or interpretations of drug resistances^28–31^. To support our protocol so far, and to provide insights on the stability of the predicted binding mode of compound 11 in the binding site of nsp16, also to identify and study the dynamic nature of the SARS COV-2 2′-O-methyltransferase (nsp16) and correlate this to the key biological role played by this enzyme in the virus life cycle, we conducted three molecular dynamic simulation experiments.

### RMSD analysis and hydrogen bond monitoring

**Figure 7.**
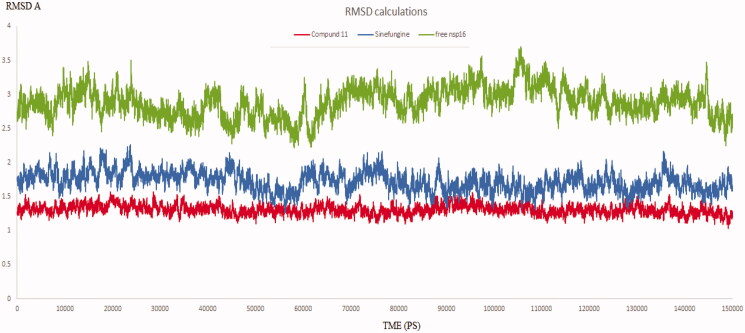
The RMSD of three dynamic simulation experiments. Green colour represents SARS COV-2 2′-O-methyltransferase without a ligand; blue line represents SARS COV-2 2′-O-methyltransferase complex with Sinefungine, and Red line represents SARS COV-2 2′-O-methyltransferase complex with compound **11**.

The ultimate endeavour for SARS-COV-2 2′-O-methyltransferase (nsp16) is to prevent the degradation of the viral RNA through the process of Cap formation as previously mentioned. Moreover, this enzyme should have a high degree of flexibility and dynamicity to deliver its intended function^32^^,^^33^. So, the conducted simulation experiment for the unbound SARS-COV-2 2′-O-methyltransferase aimed to be used as a reference for comparison with the other two simulation experiments. The calculated RMSD of all the residues of the unbound enzyme reached 3.70 A° revealing the high dynamic properties of the SARS-COV-2 2′-O-methyltransferase (nsp16), (Figure 7).

The goal of this section is to provide evidence on the stable binding of the proposed inhibitors to nsp16. Based on the proven degrees of flexibility of the binding site of nsp16, simulating the enzyme-inhibitor complex provides a consistent parameter to evaluate its stability upon binding. Thus, it was important to monitor the dynamic behaviour for both SARS-COV-2 2′-o-methyltransferase (nsp16) complex with Sinefungine, and SARS-COV-2 2′-O-methyltransferase (nsp16) complex with compound **11** through measuring the RMSD for both complexes.

RMSD values for both compound **11** and Sinefungine reached at their maximum dynamicity peaks 1.56 A° and 2.25 A°, respectively (Figure 7). This indicates that compound **11** shows better binding to SARS COV-2 2′-O-methyltransferase (nsp16) even more than Sinefungine. Also, it was worthy to monitor the stability of the interactions formed between compound **11** and SARS COV-2 2′-O-methyltransferase (nsp16) through the entire MD experiment. GROMACS has built-in commands that were used to measure the distances of the formed hydrogen bonds between compound **11** and SARS-COV-2 2′-O-methyltransferase (nsp16). The distance between the hydrogen bond donor and the hydrogen bond acceptor in a valid hydrogen bonding should be always less than 3.5 A°. This criterion was fulfilled in all the formed hydrogen bonds between compound **11** and its target indicating a stable and valid mode, (Table 2).

**Table 2.** The average distances of all the hydrogen bonds formed between compound **11** and SARS COV-2 2′-O-methyltransferase (nsp16) through the entire 150 ns MD simulation.

Hydrogen bond name	Average distance (A^0^) ± SD
Hydrogen bond with Leu6898	2.37 ± 0.22
Hydrogen bond with Tyr6930	2.41 ± 0.18
Hydrogen bond with Asn6996	1.98 ± 0.09
Hydrogen bond with Glu7001	3.11 ± 0.11

### MM-PBSA binding free energy calculations

Another important indicator that gives account for the potential affinity of a ligand with its target is the binding free energy calculated using MM-PBSA and MD calculations. In general, complexes that have lower binding free energy can be considered to be more stable and their ligands are expected to have high activity and potency. In MD simulation, the binding free energies are calculated for every conformation saved in the trajectory. Accordingly, the g_mmpbsa package was used to calculate the MM-PBSA binding free energy for the two complexes of SARS COV-2 2′-O-methyltransferase enzyme with compound **11** and with Sinefungine by the employment of MmPbSaStat.py python script. This script brings the package in action to calculate the total free energy for each component of the complex; i.e. the energy of the complex, receptor and the ligand, etc.

Furthermore, the free energy for each component could be calculated by the cumulative sum of its molecular mechanics potential energy in a vacuum and the free energy of solvation. The free energy of solvation includes the polar solvation energy (electrostatic) and nonpolar solvation energy (non-electrostatic), the last one is usually by the solvent accessible surface area (SASA) model. The g_mmpbsa package was used to calculate all those types of energies along with the values standard deviation and then summed together to yield the average total free energy of each component. Finally, the binding free energy could be calculated by subtracting the total free energy of the receptor and the total free energy of the ligand from the total free energy of the complex. Table 3 summarises the interaction energies and the binding free energy for the two complexes.

**Table 3.** The calculated interaction energies and the binding free energy for compound **11** and Sinefungine complexes with SARS COV-2 2′-O-methyltransferase.

Complex	**ΔE**_binding (kj/mol)_	**ΔE**_Electrostatic (kj/mol)_	**ΔE***_Vander Waal_*_(kj/mol)_	**ΔE**_polar solvation (kj/mol)_	**SASA**_(kJ/mol)_
Compound **11**	–296.95 ± 17.53	–102.82 ± 17.45	–263.40 ± 20.09	98.58 ± 14.92	–23.31 ± 0.99
Sinefungin	–260.86 ± 16.41	–94.13 ± 15.74	–218.08 ± 19.54	71.68 ± 13.97	–20.33 ± 1.04

Generally, compound **11**–SARS COV-2 2′-O-methyltransferase complex was better than Sinefungine complex in all the calculated energy formats except (polar solvation energy). Its average binding free energy reached −296.95 Kj/mol, while Sinefungine average binding free energy reached −260.86 Kj/mol. The overall results of the three dynamic simulations supported our concept of design and validated the entire virtual screening approach; also, they emphasised the potential inhibitory effect of compound **11** on SARS COV-2 2′-O-methyltransferase.

## Conclusion

In the current study we constructed a protocol of structure-based virtual screening aiming at identifying a novel potential inhibitor for SARS COV-2 2′-O-methyltransferase. We used the crystal structure of SARS-COV-2 2′-O-methyltransferase (PDB ID: 6WKQ) in complex with Sinefungine. First, the crystal structure was retrieved from the protein data bank. Then, a 3D pharmacophore model of 4 features and 7 components was constructed based on the binding interactions of Sinefungin with the enzyme. Thereafter, the Zinc database containing 48 Million drug-like compounds was screened through the pharmacophore model. Only 24 compounds were able to pass the pharmacophore filter and were docked subsequently into the binding site of the enzyme. Compounds (**5**, **9**, **11** and **24**), the best score-ordered hits of the docking result, were selected for further analysis since they exhibited better scores compared to Sinefungin. As a refinement step, we conducted three molecular dynamic simulation experiments for 150 ns. The MD and MM-PBSA outputs revealed compound **11** as the best potential nsp16 inhibitor herein identified, as it displayed a better stability and average binding free energy for the ligand-enzyme complex compared to Sinefungin.

## Supplementary Material

Supplemental Material
