# Supplementary material for: In silico identification of novel SARS-COV-2 2′-O-methyltransferase (nsp16) inhibitors: structure-based virtual screening, molecular dynamics simulation and MM-PBSA approaches
Source: J Enzyme Inhib Med Chem. 2021 Mar 9;36(1):727–36. doi: 10.1080/14756366.2021.1885396 (PMC7946047; doi:10.1080/14756366.2021.1885396)
Supplement: Supplemental Material [file IENZ_A_1885396_SM8724.pdf]

Supporting Information

In silico identification of novel SARS-COV-2 2′-O-methyltransferase (nsp16) inhibitors: Structure-based virtual screening, molecular dynamics simulation and MM-PBSA approaches

Mahmoud A. El Hassab*, Tamer M. Ibrahim, Sara T. Al-Rashood, Amal Alharbi, Razan O. Eskandrani, Wagdy M. Eldehna*

A.

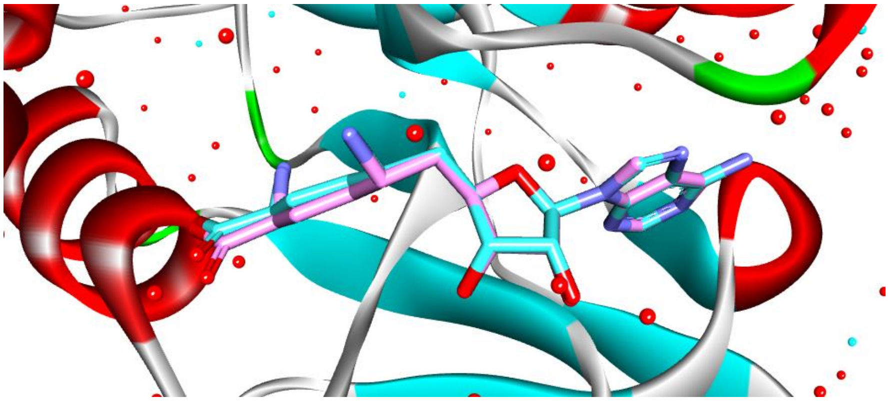

B.

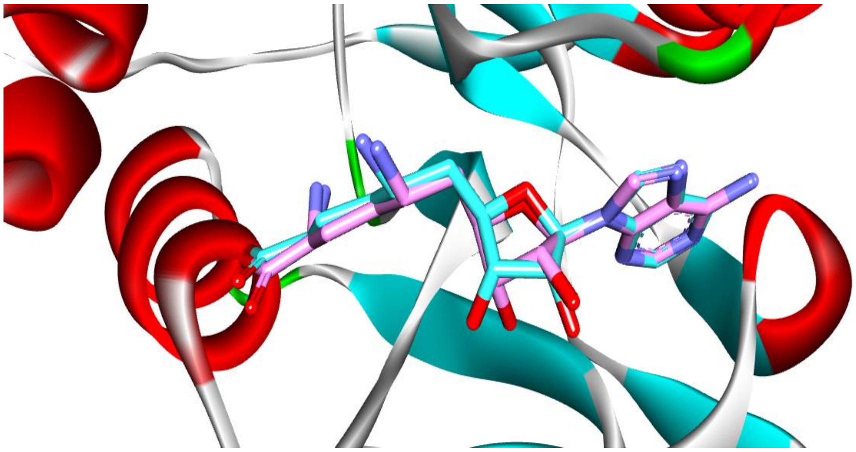

Figure S1. Superimposition between co-crystalized (Cyan) and re-docked pose (Pink) of Sinefungine (A) in presence of water (B) in absence of water showing nearly the same binding. mode.

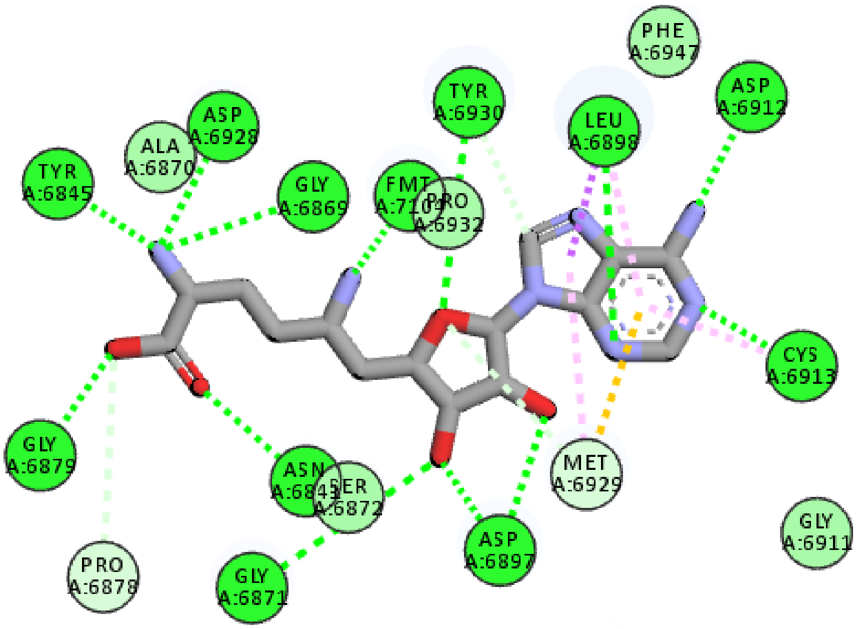

Figure S2. Binding of Sinefungine to SARS COV-2 2′-o-methyltransferase (nsp16) before equilibration showing many interactions that may be either significant or not.

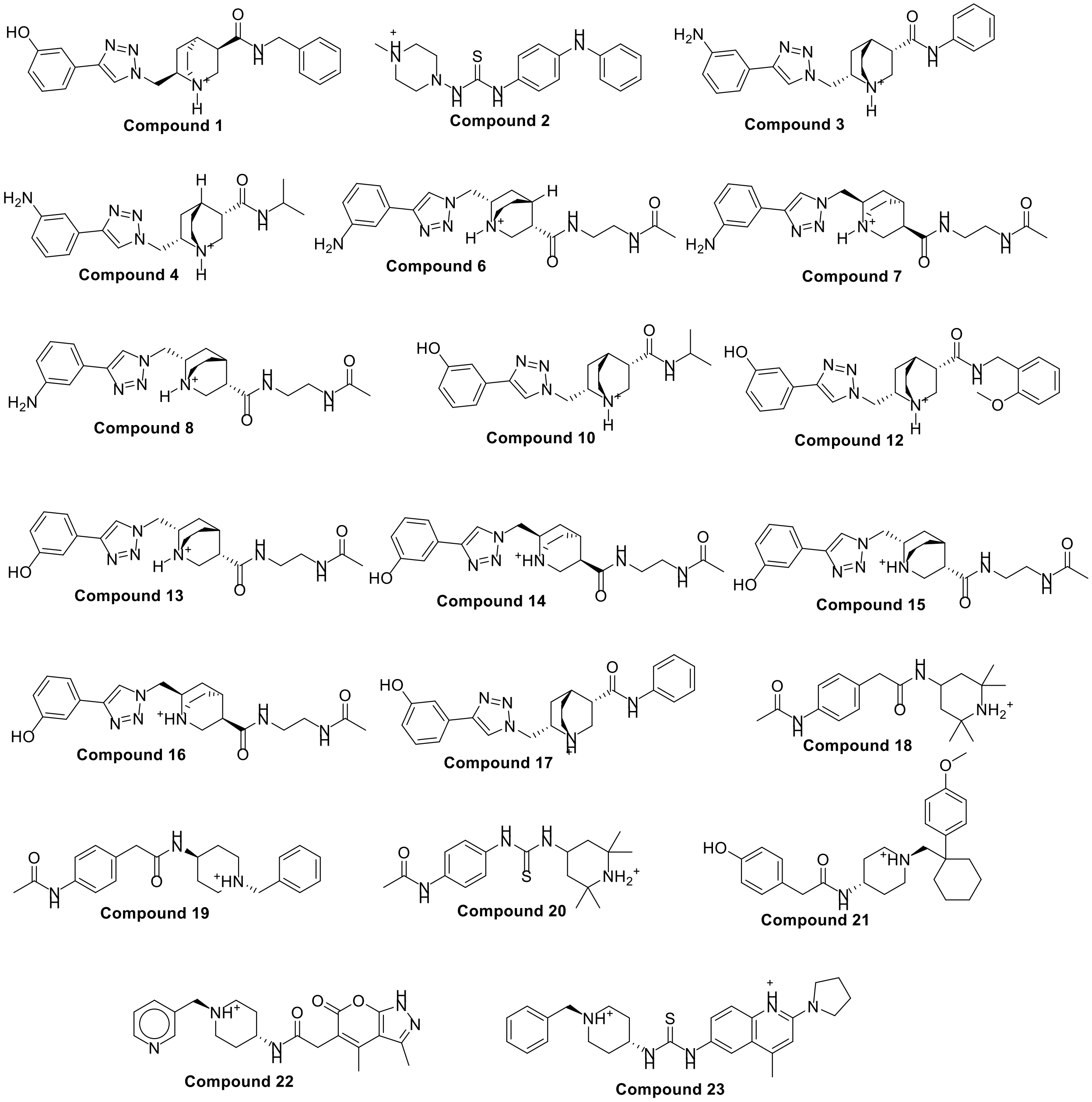

Figure S3. Chemical structures for compounds passed the pharmacophore filter and were further screened through docking-based virtual screening.
